# Lung ultrasound for the diagnosis of pneumonia in adults: A meta-analysis

**DOI:** 10.1097/MD.0000000000005713

**Published:** 2017-01-20

**Authors:** Ling Long, Hao-Tian Zhao, Zhi-Yang Zhang, Guang-Ying Wang, He-Ling Zhao

**Affiliations:** Department of Intensive Care Unit, Hebei General Hospital, Hebei, China.

**Keywords:** accuracy, diagnosis, lung ultrasound, pneumonia

## Abstract

**Background:**

Pneumonia is a common and serious infectious disease that can cause high mortality. The role of lung ultrasound (LUS) in the diagnosis of pneumonia is becoming more and more important.

**Methods:**

In the present study, we collected existing evidence regarding the use of LUS to diagnose pneumonia in adults and conducted a systematic review to summarize the technique's diagnostic accuracy. We specifically searched the Cochrane Central Register of Controlled Trials (CENTRAL), PubMed, and Embase databases and retrieved outcome data to evaluate the efficacy of LUS for the diagnosis of pneumonia compared with chest radiography or chest computed tomography. The pooled sensitivity (SEN) and specificity (SPE) were determined using the Mantel–Haenszel method, and the pooled diagnostic odds ratio (DOR) was determined using the DerSimonian–Laird method. We also assessed heterogeneity of sensitivity, specificity, and diagnostic odds ratio using the *Q* and *I*^2^ statistics.

**Results:**

Twelve studies containing 1515 subjects were included in our meta-analysis. The SEN and SPE were 0.88 (95% CI: 0.86–0.90) and 0.86 (95% CI: 0.83–0.88), respectively. The pooled negative likelihood ratio (LR) was 0.13 (95% CI: 0.08–0.23), the positive LR was 5.37 (95% CI: 2.76–10.43), and the DOR was 65.46 (95% CI: 29.24–146.56). The summary receiver operating characteristic curve indicated a relationship between sensitivity and specificity. The area under the curve for LUS was 0.95.

**Conclusion:**

LUS can help to diagnose adult pneumonia with high accuracy.

## 1 Introduction

Pneumonia is a frequent disease in adults, representing a major healthcare and economic problem. Despite improvement in patient management, community-acquired pneumonia (CAP) remains a leading cause of worldwide. The global annual incidence of CAP in children remains at 150 to 156 million cases, and 922 thousands are killed by this condition each year.^[1]^ Annually, 15 people per 1000, and predominantly the young and elderly, visit a doctor for symptoms of CAP.^[2]^ At least 20% of CAP patients require hospitalization, 25% of whom need treatment in an intensive care unit (ICU), and the mortality rate is 30 to 50%.^[3]^ In European countries, the median estimated cost of the median length of stay ranges from €1200 to €6900.^[4]^ Due to the clinical and the financial burdens of CAP, efficient and cost-effective diagnostic options for pneumonia are an important determinant of hospital costs and efficiency.

The diagnosis of pneumonia was once thought to be confirmable simply by physical examination, history taking, and related methods. However, signs and symptoms cannot provide certainty about this diagnosis that needs an imaging examination to confirm. Chest radiography is recommended as the main imaging approach for diagnosing pneumonia. However, there must be a strong of the differential diagnosis of pneumonia pathology and chest computed tomography (CT) is considered the gold-standard imaging approach. However, limitations on CT use also exist, as it is always troublesome that this method has a high cost and a high radiation exposure dose.^[5]^ Particularly among unstable critically ill patients, chest radiography and CT are not adequately easy to use. Recently, lung ultrasound (LUS), which is currently being used as a bedside method, has been shown to be useful for evaluating a range of pathologic pulmonary conditions.^[6]^ In fact, LUS has better sensitivity than chest radiography in pleural effusion diagnosis.^[7]^ In addition, several studies have demonstrated the application of ultrasound in the diagnosis of pneumonia, and the results have been significant.^[8]^ In contrast to a recent meta-analysis^[9]^ that used chest CT as the only reference standard, our review sought to evaluate the diagnostic accuracy of LUS in pneumonia in more studies; the reference standards in those studies were not only chest CT but also chest radiography.

## 2 Method

### 2.1 Study identification

The Cochrane Central Register of Controlled Trials (CENTRAL), PubMed, and Embase databases were searched up to October 2015 for relevant studies in English. We included published and ongoing trials and used a systematic search strategy in collaboration with 2 investigators. We specifically implemented the PubMed search strategy using the terms listed in Table 1. Our study does not require the approval of the ethics committee.

**Table 1:**
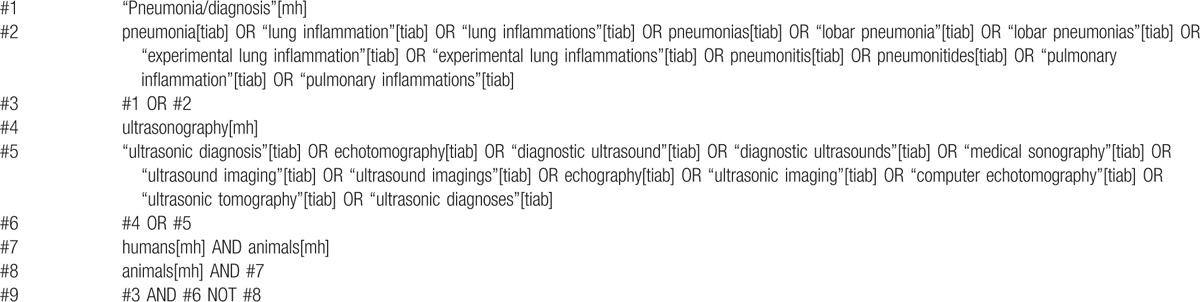
Pubmed search strategy for meta-analysis.

### 2.2 Selection of studies and criteria of eligibility

Two authors independently evaluated all studies for relevance using the search strategy at the title, abstract and full-text levels. Disagreements were resolved by a third author. We pursued all studies using the following inclusion criteria: enrollment of patients aged 18 years or above, enrollment of patients with clinical suspicion of pneumonia, and a reference method for diagnosing pneumonia that was based on respiratory signs and symptoms and confirmation by chest radiography or chest CT. We excluded studies that enrolled children^[10]^ and studies that evaluated pneumonia only based on clinical data. Two reviewers independently evaluated all relevant studies based on the eligibility criteria, and a study was excluded if it did not meet the above-mentioned criteria.

### 2.3 Data extraction and quality assessment

Two authors independently extracted data via a standardized form, including data on the fundamental characteristics of the studies and their outcomes. The fundamental characteristics included the name of the first author, publication year, study design, mean age of the study population, sample size, and male/femal percentage. The outcomes were the numbers of true positives, true negatives, false positives, and false negatives. The data that were collected from each study were evaluated using the Quality Assessment of Diagnostic Accuracy Studies (QUADAS-2) criterion.^[11]^ This standardized approach rates the quality of studies included in systematic reviews of diagnostic accuracy.

### 2.4 Data synthesis and analysis

The primary aim of our meta-analysis was to estimate the accuracy of diagnostic measurements. Within the context of a bivariate regression approach, we estimated the pooled sensitivity and specificity using the Mantel–Haenszel method and the pooled positive and negative likelihood ratios (LRs) using the DerSimonian–Laird method.^[12]^ A summary receiver operating characteristic (SROC) curve was also constructed to summarize the study results.^[13]^ The significance of the between-study heterogeneity and variance was evaluated by the test of inconsistency (*I*^2^) of the pooled diagnostic odds ratio (DOR).^[14]^ The DOR and its relevant 95% CI were specifically pooled by using fixed-effect or random-effect models. In particular, an *I*^2^ value less than 50% indicated a lack of heterogeneity among the studies, in which case the pooled DOR was calculated using the fixed-effect model; otherwise, the random-effect model was used.^[15]^ Publication bias was estimated with a visual inspection of funnel plots. All of the statistical analyses were performed using RevMan 5.3 or Meta-DiSc 1.4 (Ramony Cajal Hospital, Madrid, Spain).

## 3 Results

**Figure 1:**
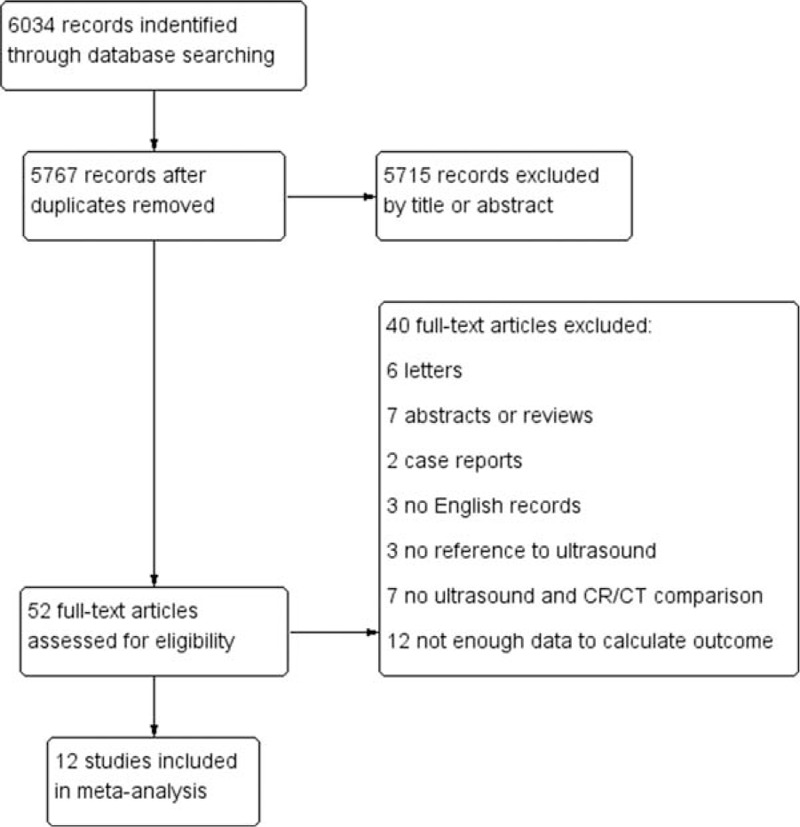
Flowchart of articles retrieved from the search of databases and reasons for exclusion.

**Figure 2:**
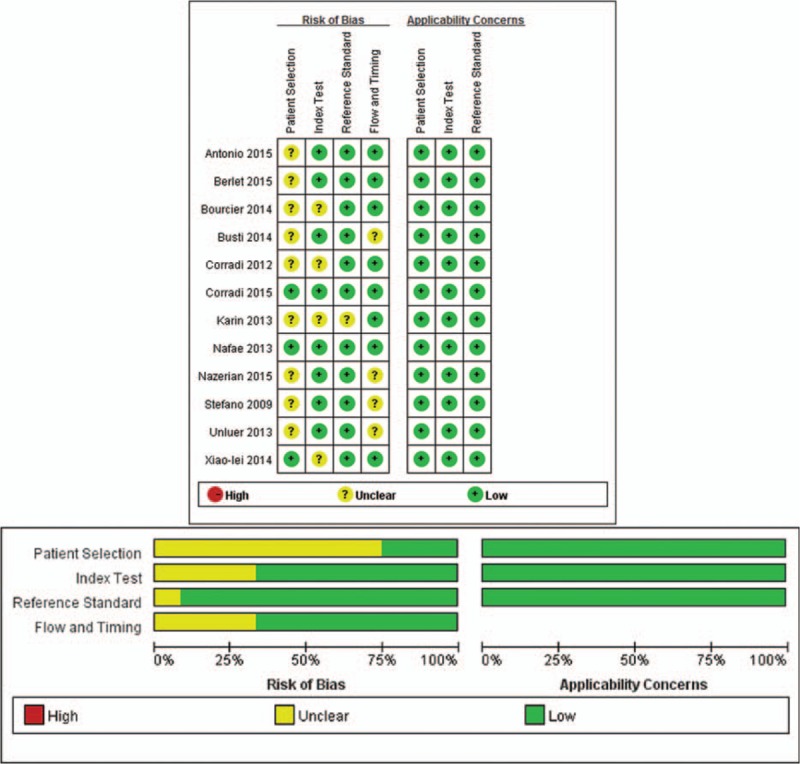
Details of quality assessment by the QUADAS-2 tool.

**Figure 3:**
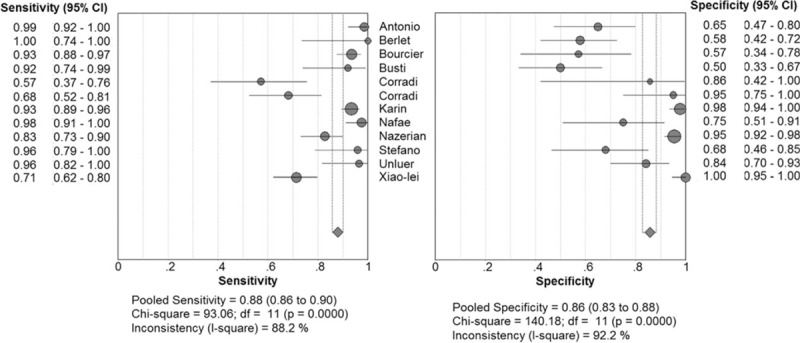
Forrest plots of pooled sensitivity and specificity.

**Figure 4:**
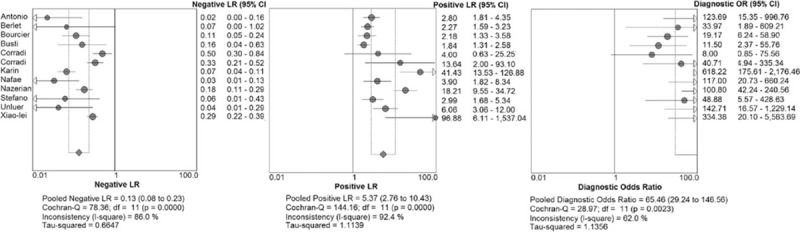
Forest plots for lung ultrasound for the diagnosis of pneumonia.

**Figure 5:**
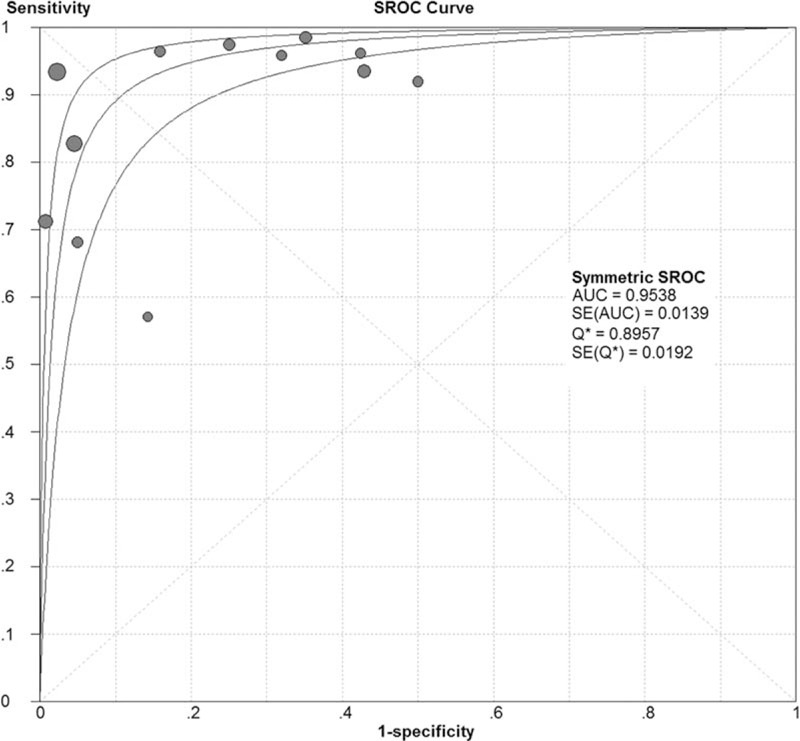
The summary receiver operating characteristic of LUS for the detection of pneumonia compared with CR or chest CT. CT = computed tomography.

We identified 6034 studies that fit our search strategy, 12 of which fulfilled the inclusion criteria and were included in our analysis, as shown in Fig. 1 and Table 2.^[16–27]^ The results of this assessment are given in the “risk of bias summary” in Fig. 2. These studies included 1515 subjects who were randomized to chest radiography or chest CT prior to LUS. The chest radiography or chest CT was used as a reference standard for pneumonia diagnosis in our review. There were 860 cases of pneumonia and 655 with no pneumonia. Four studies used chest CT as the reference standard in the whole sample,^[18,19,21,24]^ Six studies used chest radiography for diagnosis in all subjects,^[16,17,20,22,23,26]^ and 2 studies used either chest CT or chest radiography as the reference standard.^[25,27]^ The overall pooled SEN and SPE were 0.88 (95% CI: 0.86–0.90) and 0.86 (95% CI: 0.83–0.88), respectively (Fig. 3). The calculated pooled negative LR was 0.13 (95% CI: 0.08–0.23), the positive LR was 5.37 (95% CI: 2.76–10.43), and the DOR was 65.46 (95% CI: 29.24–146.56) (Fig. 4). The SROC curve indicated a relationship between sensitivity and specificity. The area under the curve (AUC) for LUS was 0.95 (Fig. 5), indicating the highly discriminatory ability of LUS.

**Table 2:**
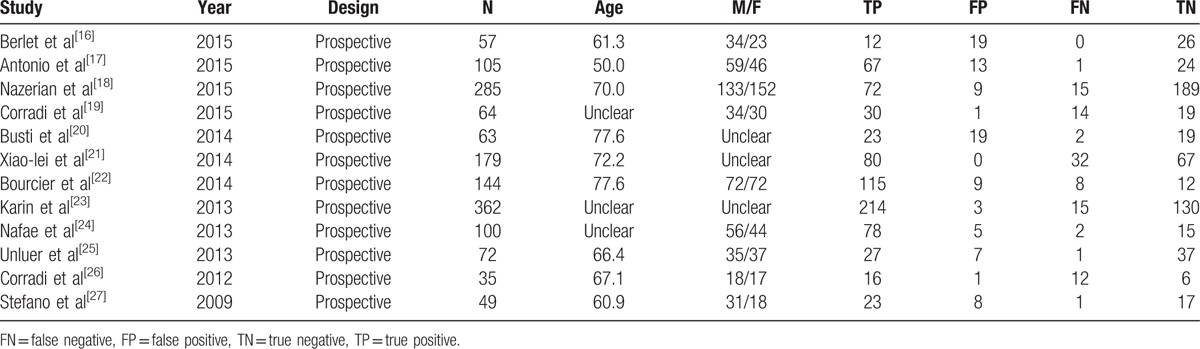
Fundamental characteristics from the 12 studies.

**Figure 6:**
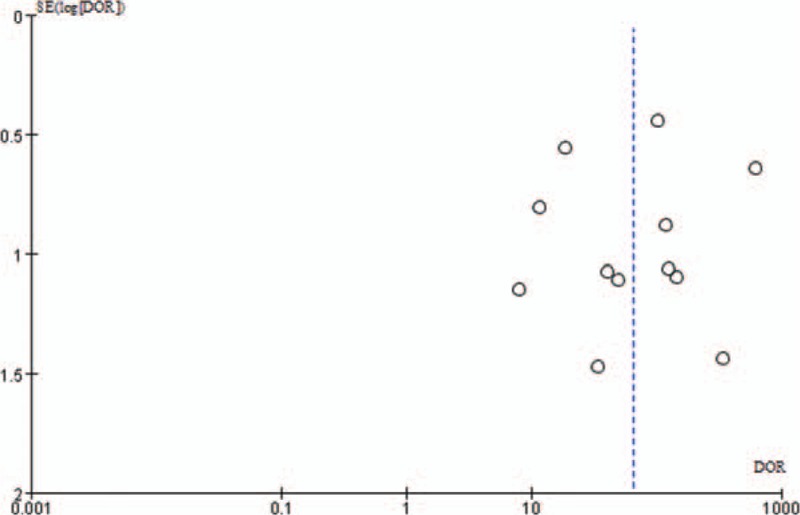
Funnel plot.

The funnel plot appeared relatively symmetrical, as shown in Fig. 6. But there only a limited number of studies were included in this meta-analysis.

## 4 Discussion

The diagnosis of pneumonia has recently become highly dependent on chest X-ray or chest CT. However, it is difficult to distinguish between pneumonia and pulmonary embolism, acute respiratory distress syndrome, and pulmonary fibrosis. Over the last 20 years, ultrasound has been shown to be highly effective in evaluating a range of pathologic pulmonary conditions.^[28]^ In our meta-analysis, we found that compared with chest radiography or chest CT, LUS had high SEN (88%) and SPE (86%) for the detection of adult pneumonia. In 2014, Bourcier et al^[22]^ also noted the diagnostic value of LUS in pneumonia. This study specifically revealed a significantly higher sensitivity of LUS for the diagnosis of acute pneumonia compared with to chest radiography (95% vs 60%, *P* >0.01). Moreover, when chest CT was performed due to difficult diagnosis, the performance of LUS in acute pneumonia diagnosis was 100% in comparison.

In the present analysis, based on the sensitivity and specificity, we could calculate the DOR, which is a single indicator of test accuracy.^[29]^ The AUC was 0.95, indicating a high level of overall accuracy. The DOR is the ratio of the positive LR relative to the negative LR, and the higher the DOR is, the greater the accuracy of the method for the diagnosis for pneumonia. In our review and meta-analysis, the mean DOR was 65.46 (95% CI: 29.24 to 146.56), showing a significantly high level of overall accuracy. However, LRs are more clinically meaningful. The pooled positive LR of 5.37 suggests that patients with pneumonia have a 5- to 6-fold higher chance of being LUS positive compared with patients without pneumonia. In contrast, the pooled negative LR of 0.13 suggests that if an LUS is negative, the probability that the patient has pneumonia is 13%.^[30]^ A meta-analysis conducted by Chavez et al^[31]^ that considered clinical manifestations, laboratory results, and chest imaging by chest radiography or chest CT as the diagnostic criteria identified a clear advantage of LUS over standard imaging for pneumonia, with a positive LR, negative LR, and AUC of 16.8 (95% CI: 7.7–37.0), 0.07 (95% CI: 0.05–0.10), and 0.98 (95% CI: 0.98–0.99), respectively. Moreover, LUS can be performed in less than 13 minutes.

With the development of ultrasonic technology, more and more experiments have demonstrated the value of ultrasound in the diagnosis of pneumonia. Regarding the accuracy of LUS for the diagnosis of pneumonia, a meta-analysis conducted by Chavez et al was published in 2014.^[31]^ Our meta-analysis is similar to the above review. But there are certain differences between this meta-analysis and ours. First, the diagnostic criteria of pneumonia in studies that were selected by Chavez et al is clinical diagnosis or imaging. Our meta-analysis evaluated pneumonia based on clinical signs and symptoms and used either chest radiography or chest CT as the reference standard. Second, Chavez et al included fewer studies. We have more recent studies and which were almost published in 2014 and 2015. Finally, because our inclusion criteria are stricter, the prior analysis had higher sensitivity. So, the overall accuracy was relatively lower in our meta-analysis, but it still identified LUS as a reliable tool for diagnosing pneumonia.

LUS is a simple method that avoids the use of radiation. In addition, LUS can be used explores certain findings that are not obvious on chest radiography.^[32]^ In the present review and meta-analysis, certain limitations should be considered. First, we only included articles in English. Second, not all studies used the same gold standard. Third, the ability of operators to perform LUS was not analyzed and the accuracy of LUS in diagnosis of pneumonia depended on the skills of the operators. Fourth, not all lung regions were assessed. Fifth, the number of studies in our analysis was relatively small, and these studies may not have adequately assessed the diagnostic accuracy. Thus, more clinical studies are needed to further investigate the diagnostic accuracy of LUS in pneumonia.

## 5 Conclusion

Our meta-analysis indicates that LUS has high accuracy in the diagnosis of pneumonia. Moreover, LUS has bedside availability and feasibility. Therefore, LUS may be a promising alternative to chest X-ray and chest CT when it is not inconvenient.
